# Effect of a music intervention on anxiety in adult critically ill patients: a multicenter randomized clinical trial

**DOI:** 10.1186/s40560-023-00684-1

**Published:** 2023-08-17

**Authors:** Ellaha Kakar, Thomas Ottens, Susanne Stads, Sanne Wesselius, Diederik A. M. P. J. Gommers, Johannes Jeekel, Mathieu van der Jagt

**Affiliations:** 1 Department of Intensive Care, Erasmus MC, University Medical Center, Doctor Molewaterplein 40, Room NA-2123, 3015 GD Rotterdam, The Netherlands; 2 Department of Surgery, Erasmus MC, University Medical Center, Rotterdam, The Netherlands; 3 Department of Intensive Care, Haga Teaching Hospital, The Hague, The Netherlands; 4 Department of Intensive Care, Ikazia Hospital, Rotterdam, The Netherlands; 5 Department of Neuroscience, Erasmus MC, University Medical Center, Rotterdam, The Netherlands

**Keywords:** Music, Intensive care unit, Anxiety, Non-pharmacologic intervention

## Abstract

**Background:**

Previous studies show positive effect of music on reducing anxiety, pain, and medication requirement. Anxiety has become a more pertinent issue in the intensive care unit (ICU) since wakefulness is preferred according to recent guidelines. Nevertheless, evidence on the effect of music in ICU patients is scarce. Therefore, we studied the effect of music intervention on anxiety in ICU patients.

**Methods:**

A multicenter randomized clinical trial was conducted between August 2020 and December 2021 in ICU’s at an academic medical centre and two regional hospitals. Adult critically ill patients were eligible when hemodynamically stable and able to communicate (Richmond agitation-sedation scale (RASS) of at least − 2). Patients in the intervention arm were offered music twice daily during three days for at least 30 min per session. Patients in the control group received standard care. The primary outcome was anxiety level assessed with the visual analogue scale for anxiety [VAS-A; range 0–10] twice daily (morning and evening). Secondary outcomes included; 6-item state-trait anxiety inventory (STAI-6), sleep quality, delirium, heart rate, mean arterial pressure, pain, RASS, medication, ICU length of stay, patients’ memory and experience of ICU stay.

**Results:**

94 patients were included in the primary analysis. Music did not significantly reduce anxiety (VAS-A in the intervention group; 2.5 (IQR 1.0–4.5), 1.8 (0.0–3.6), and 2.5 (0.0–3.6) on day 1, 2, and 3 vs. 3.0 (0.6–4.0), 1.5 (0.0–4.0), and 2.0 (0.0–4.0) in the control group; *p* > 0.92). Overall median daily VAS-A scores ranged from 1.5 to 3.0. Fewer patients required opioids (21 vs. 29, *p* = 0.03) and sleep quality was lower in the music group on study day one [5.0 (4.0–6.0) vs. 4.5 (3.0–5.0), *p* = 0.03]. Other outcomes were similar between groups.

**Conclusions:**

Anxiety levels in this ICU population were low, and music during 3 days did not decrease anxiety. This study indicates that efficacy of music is context and intervention-dependent, given previous evidence showing decreased anxiety.

*Trial registration* Netherlands Trial Register: NL8595, Registered, 1 April 2020. ClinicalTrials.gov ID: NCT04796389, Registered retrospectively, 12 March 2021

**Supplementary Information:**

The online version contains supplementary material available at 10.1186/s40560-023-00684-1.

## Background

Anxiety is common in intensive care unit (ICU) patients and occurs in 30–80% of patients [1–3]. However, routine assessment of anxiety is variable [4]. Anxiety in the ICU not only reduces patient comfort, but can also have behavioural and physiological consequences, e.g. through elevated stress level [4–6]. Furthermore, anxiety and pain are strongly correlated, and may reinforce each other leading to higher sedatives and analgesics requirements [2, 7]. These medications are known to have negative side effects, such as prolonged mechanical ventilation [8–13]. Currently, there are limited therapeutic options for anxiety other than analgo-sedation and there are no clear guideline recommendations for non-pharmacologic treatment of anxiety in the ICU [4]. The Clinical Practice Guidelines for the Prevention and Management of Pain, Agitation/Sedation, Delirium, Immobility, and Sleep Disruption in Adult Patients in the ICU (PADIS) strongly recommend avoiding sedatives, especially benzodiazepines, whenever possible due to negative side effects. In addition, the tendency to strive for wakefulness in ICU patients may add to the incidence and severity of anxiety.

Music may be a useful treatment to alleviate anxiety. Music as a non-pharmacologic therapy has been widely studied and has shown beneficial effects in other settings, e.g. on perioperative anxiety and pain and neurohormonal stress response [14, 15]. Other studies suggested positive effects of music interventions in the ICU on pain, anxiety, stress, and sedatives, and analgesics medication requirements [6, 16–21]. An additional advantage of music is that it is probably risk-free, since no risks have been described in current literature. A previous randomized controlled trial by Chlan et al. [20] evaluated the effect of patient-directed music intervention on anxiety in the ICU and reported a positive effect. However, it is likely that efficacy of the intervention is highly context specific and therefore may not be reproducible in other settings.

Since anxiety may be under-detected but is burdensome for patients, there is a need for effective non-pharmacologic interventions that are widely applicable and effective [3, 7]. Based on the previous, we hypothesize that music may positively influence anxiety, but since research on the effect of music on anxiety in wakeful ICU patients is scarce [19], we studied the effect of a music intervention on anxiety in critically ill patients.

## Methods

### Study design

This multicenter, randomized, controlled trial was conducted between August 2020 and December 2021 and took place at the ICU’s of one academic and two tertiary referral hospitals in the Netherlands. The study was approved by the Medical Ethics Review Board of Erasmus MC (MEC2020-0212) and the local institutional review boards (Ikazia Hospital: IZ/705/SW2037, Haga Teaching Hospital: T20-080). The trial was registered in the Netherlands Trial Register (www.trialregister.nl, ID: NL8595) and the United States National Library of Medicine (www.clinicaltrials.gov, ID: NCT04796389). The study protocol has been previously published [22]. The study is reported according to the Consolidated Standards of Reporting Trials (CONSORT) 2010 statement (Additional file 1) [23].

### Study population

ICU patients aged 18 years or older, either mechanically ventilated or not, were eligible for inclusion in the study when meeting the following criteria: hemodynamically stable (as assessed by the patient’s direct caregivers), able to communicate (Richmond Agitation and Sedation Scale, RASS > − 3 in the 24 h before inclusion (meaning the patient was at least briefly awakened with eye contact to voice) and was considered to be able to provide information regarding anxiety level, had an expected ICU stay upon randomization of at least 48 h, and a written informed consent was acquired from the patient or legal representative. Exclusion criteria were: severe hearing impairment, neurological condition (e.g. severe stroke, when deemed to interfere with processing of music), insufficient knowledge of the Dutch or English language, and participation in another study that may possibly intervene with the primary outcome (level of anxiety).

### Randomization and masking

Parallel block randomization was used to allocate subjects with an equal allocation ratio in either the intervention or the control group using online web-based randomization program. Subjects were equally allocated by centre. In order to prevent bias due to non-blinding of the outcome assessors (member of the research team or attending nurse), the patient reported outcomes were accompanied by a clear description of how they should be assessed.

### Intervention

Richard-Lalonde et al. [21] found that music interventions of at least 20–30 min significantly reduced pain scores compared to 10–15 min in critically ill patients [21]. Furthermore, Chlan et al. and Fu et al. [24, 25] found that a total of 80–120 min per day music intervention leads to significant reduction in anxiety and sedative and analgesic medication requirement [20, 25]. In addition, several studies suggest the importance of individual music preference of ICU patients in the effectiveness of the music intervention [16, 21]. Based on the previous studies, subjects allocated to the intervention arm were offered to listen to music during three days twice per day, in the morning and evening, during at least 30 min per session in addition to standard care. The intervention was applied as mentioned in order to restore the circadian cycle of the patient, since this is known to be disturbed, by offering music after awakening in the morning and before the lights were turned out in the evening [4]. Music intervention was provided through over-the-ear headphones connected through Bluetooth with a tablet on which a large variety of online music lists were available (based on genre/artist/type, etc., which were pre-arranged or could be re-arranged by investigator, nurse of the patient), from which the patients’ preferred music could be chosen. Music preference was assessed by the patients, or legal representative if the patient was not able to do so, family members, or friends at baseline directly after inclusion and randomization (day before the start of the intervention). We discouraged patients to listen to rock and heavy metal music during the trial, since it is likely that loud and/or rock music may lack the right qualities for this setting [26]. The first session was planned in the morning, between 09.00 and 12.00 AM, the day after inclusion. The evening session was planned before intended sleep, generally between 20.00 and 23.00 PM. In agreement with the direct caregivers, patients were allowed to listen more often or longer to music as requested by the patient or legal representative. Music was only provided when patients were conscious and could reply to the question whether they wanted to listen to music. Additionally, we encouraged nurses to document music being played apart from the music applied with the headphones within the trial protocol, although this was discouraged. Patients in the control group received standard care (no changes in routine care) without structured music intervention.

### Outcomes

The primary outcome was level of anxiety as assessed with the visual analogue scale for anxiety (VAS-A), which was assessed directly after the music session in the morning and evening. The VAS-A is validated as a reliable self-rating tool for state anxiety and has been used in the intensive care setting [19, 20, 27]. The VAS-A is a patient reported outcome and ranges from zero to ten, whereas zero is defined as “no feeling of anxiety” and ten as “most anxious ever” on a horizontal scale. The effect of music on anxiety was also assessed using the six-item State-Trait Anxiety Inventory (STAI-6, which only assesses state anxiety, and was added as an additional tool since it assesses anxiety dimensions, such as anxiety about an event, or anxiety level as a personal characteristic). The sum score of the STAI-6 ranges from 20 to 80 and was categorized as low (score of 20–39), moderate (score of 40–59), or high anxiety level (score of 60–80) [28, 29]. Furthermore, we assessed sleep quality (with a Visual Numeric Scale ranging from one to seven, in which one indicates “did not/barely sleep” and seven indicates “slept very well”) [30], pain (using the Critical care Pain Observation Tool (CPOT) in mechanically ventilated patients and the numeric rating scale (NRS)/VAS for pain in non-ventilated) [4], medication requirement (analgesics, sedatives, and antipsychotics, reported as daily administration [yes/no] and dosages), RASS, delirium (measured with the Intensive Care Delirium Screening Checklist [ICDSC]), complications related to agitation, including auto-removal of lines and tubes, time on mechanical ventilation, ICU LOS, physical parameters (heart rate [HR], and mean arterial pressure [MAP] at the time anxiety assessments, and patients’ ICU memory and experiences. Memory was evaluated with the ICU memory tool (ICUMT) [31], which we adapted and shortened to a seven-item questionnaire to avoid overlap with assessment of anxiety or delirium, and with other tools. The patient experience was evaluated in the music group using a five-item and for the control group a three-item self-made questionnaire. Justification and assessment of the tools mentioned above are described in the previously published protocol paper [22].

### Statistical analysis

The baseline characteristics were summarized using means/median (SD/IQR) and number (percentage) for continuous and categorical variables, respectively. Non-normally distributed continuous data were analysed using the non-parametric Mann–Whitney *U* test, outcomes were presented as median and interquartile ranges (IQR). Normality was assessed with the Shapiro–Wilk test and graphically in Q–Q plots. A sample size of 52 per group was needed to detect a 1.95 point difference in VAS-A, between the groups with a power of 80%, a two-sided alpha of 0.05, and a dropout rate of 10%, which was based on a previous trial [20]. Data analysis was performed using an intention to treat (ITT) approach for all patients who had at least one VAS-A assessed. The total median of the VAS-A was calculated separately for each study day. A two-sided *p*-value of < 0.05 was considered statistically significant. Our primary outcome, the median VAS-A, was analysed for days one to three separately. A multilevel linear regression with random intercepts was used to compare the change in anxiety over the three study days [32]. In the two-level linear mixed models design (multilevel linear regression model), study day was set at level one, and subjects at level two. Age and sex were included as independent variables in the model. Secondary, a per-protocol analysis was performed. The secondary outcomes were analysed using similar statistical strategy as the primary outcome. Opioid dosages were adapted into fentanyl equivalents (fentanyl intravenous (iv) + remifentanil iv [33] + (fentanyl patch/2.4) [34] + (sufentanil iv/10) [35] + (morphine iv/100) [34] + (oxycodone oral/150) [34]) and intermittent sedatives (benzodiazepines) as lorazepam equivalents (lorazepam + (temazepam/10) [36] + (oxazepam/15) [36] + (diazepam/5) [33] + (bromazepam/5) [37] + (zopiclone/3.75) [33]). Also, each STAI-6 item was analysed separately.

## Results

**Fig. 1:**
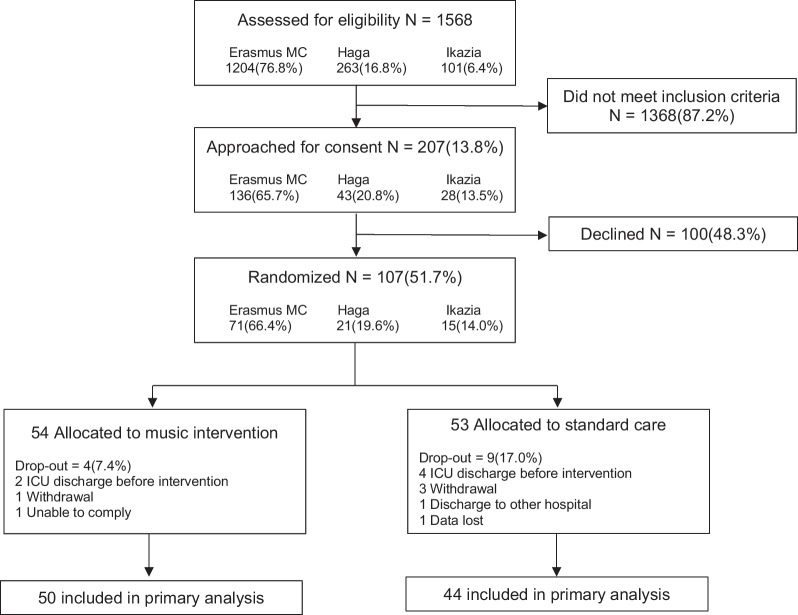
Participant flowchart

Between August 2020 and October 2021, 1568 (note: in the Erasmus MC where most of the patients were screened and included (1204 patients), contagious COVID patients, which were prevalent during the trial, were not included) patients were assessed for eligibility, of whom 207 met eligibility criteria (Fig. 1). Written informed consent was obtained from 107 patients, of whom 54 were allocated to the intervention group and 53 to the control group. The final analysis compromised 50 patients in the intervention group and 44 patients in the control group. Baseline characteristics are presented in Table 1. No differences were found in baseline characteristics between the two groups. Patients had a mean age of 62.8 ± 10.3 years, were predominantly male (66%), and had mean Acute Physiology and Chronic Health Evaluation (APACHE) IV score of 62.9 (29.9).

**Table 1:** Baseline characteristics

Characteristic	*N*	Control	*N*	Intervention	*p*
Age, years, mean (SD)	44	62.9 (9.1)	50	62.6 (11.3)	0.91
Male, %	28	63.6	34	68.0	0.82
Weight, kg, mean (SD)	44	89.5 (26.4)	50	87.1 (20.5)	0.62
Reasons for admission					
Medical	37	84.1	40	80.0	0.95
COVID-19^a^	15	34.1	18	36.0	
Surgical	6	13.6	9	18.0	
Trauma	1	2.3	1	2.0	
Comorbidities, %					
Psychiatric^b^	4	9.1	3	6.0	0.86
Chronic pain^c^	3	6.8	5	10.0	0.86
Cardiovascular	27	61.4	25	50.0	0.27
Neurologic (cerebral)	6	13.6	6	12.0	0.81
Gastro-intestinal	10	22.7	15	30.0	0.43
Hospital admission duration before inclusion, days, median (IQR)	44	17.0 (6.0–33.5)	50	16.5 (7.3–34.0)	0.72
ICU admission duration before inclusion, days	44	11.0 (3.0–28.0)	50	8.0 (3.0–29.3)	0.78
Mechanical ventilation at baseline^d^, %	44	77.3	50	72.0	0.73
Pain at baseline, median (IQR)*	33	0.0 (0.0–0.0)	44	0.0 (0.0–0.4)	0.70
ICDSC at baseline, median (IQR)	41	1.0 (0.5–2.5)	46	1.3 (0.4–2.5)	0.89
Delirium at baseline, %	7	15.9	10	20.0	0.81
APACHE IV, mean (SD)	42	61.8 (25.1)	49	64.0 (33.7)	0.72
RASS at baseline, median (IQR)	40	0 (− 1–0)	48	0 (− 1–0)	0.57
Sleep at baseline, median (IQR)*	43	5.0 (3.0–6.0)	49	4.5 (3.5–6.0)	0.81

*SD* standard deviation, *ICU* intensive care unit, *IQR* interquartile range, *ICDSC* Intensive Care Delirium Screening Checklist, *APACHE* Acute Physiology And Chronic Health Evaluation, *RASS* Richmond Agitation-Sedation Scale

^a^No differences in number of COVID-19 patients per group (*p* = 1.0)

^b^Psychiatric history: depression, anxiety, substance abuse

^c^Chronic pain history: migraine, critical illness neuropathy, plexus brachialis neuritis, hernia nucleus pulposus, problems neck for which specialized pain management is required, carpal tunnel syndrome, Bell’s paralysis

^d^Baseline is defined as day 0, the day before the intervention started

^*^Pain was assessed with pain (using the Critical-Care Pain observation (CPOT) in mechanically ventilated patients and the NRS/VAS for pain in non-ventilated)

Sleep was assessed with a Visual Numeric Scale ranging from one to seven, in which one indicates “did not/barely sleep” and seven indicates “slept very well”

### Primary outcome

**Fig. 2:**
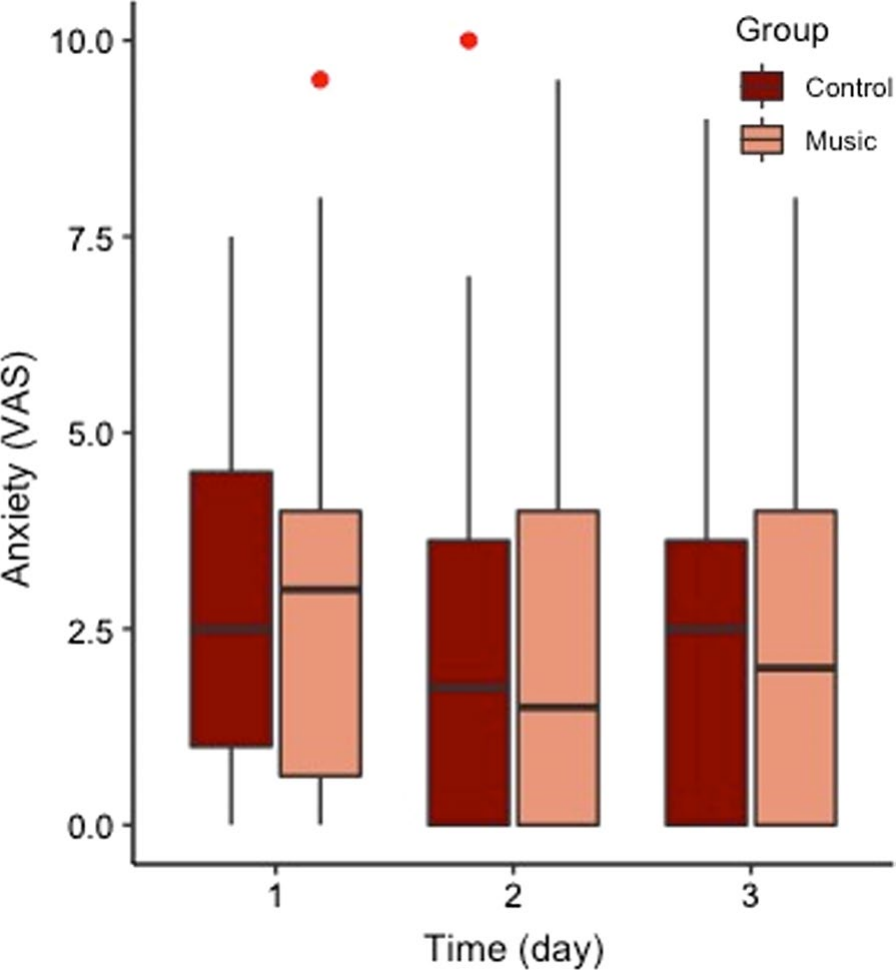
Median (IQR) anxiety scores per group

On average patients listened 49.2 ± 43.1 min of music per day (day 1; 64.1 ± 81.7 min, day 2; 45.7 ± 45.8 min, day 3; 37.9 ± 55.8 min) in the intervention group. The median (IQR) VAS-A scores in the intervention group of 2.5 (1.0–4.5), 1.8 (0.0–3.6), and 2.5 (0.0–3.6) on, respectively, day one, two, and three were similar to the VAS-A scores of 3.0 (0.6–4.0), 1.5 (0.0–4.0), and 2.0 (0.0–4.0) in the control group for both the intention to treat and per protocol analyses (Table 2, Fig. 2). Also, no significant effects were found in the mixed linear regression analysis (Additional file 2, Additional file 3).

**Table 2:** Primary outcomes

Outcome	*N*	Overall median/IQR	*N*	Control median/IQR	N	Intervention median/IQR	*p* value
Primary outcomes							
Intention-to-treat analysis							
VAS-A day 1	94	3.0 (1.0–4.5)	44	2.5 (1.0–4.5)	50	3.0 (0.6–4.0)	0.92
VAS-A day 2	85	1.5 (0.0–4.0)	40	1.8 (0.0–3.6)	45	1.5 (0.0–4.0)	0.98
VAS-A day 3	75	2.0 (0.0–4.0)	36	2.5 (0.0–3.6)	39	2.0 (0.0–4.0)	0.94
Per-protocol analysis							
VAS-A day 1	64	2.8 (1.0–4.5)	42	2.8 (1.0–4.5)	22	2.8 (0.6–4.4)	0.77
VAS-A day 2	56	2.0 (0.4–3.5)	37	2.0 (0.0–4.0)	19	1.0 (0.5–3.0)	0.42
VAS-A day 3	52	2.5 (0.4–4.0)	36	2.5 (0.0–3.6)	16	2.8 (0.9–4.5)	0.41

### Secondary outcomes

On the first study day patients in the control group reported a significantly higher quality of sleep than patients in the intervention group [median (IQR); 5.0 (4.0–6.0) vs. 4.5 (3.0–5.0), *p* = 0.03, Additional file 4]. No other significant differences were found in the secondary outcomes. Subgroup analysis of patients on mechanical ventilation did not change the results (Additional file 7).

### Medication requirement

No differences were found between the intervention and control group for continuous intravenous sedatives, intermittent sedatives, and antipsychotic requirement (Additional file 5). Only on the first study day, less patients in the intervention group used opioids (21 vs. 29, *p* = 0.03). No differences were found between the groups for fentanyl equivalents dosages. Only two patients, one in each group, had required epidural analgesia and s-ketamine, therefore further analysis was not performed for these medications.

### Complications related to agitation

Complication rates were similar between the intervention and control groups (Additional file 4).

### Follow-up: ICU memory and experience

Follow-up was done in 64 patients, 32 in each group (Additional file 6); 20 patients died, three patients withdrew consent, six were lost to follow-up, and one patient was still admitted to the ICU at the moment of this analysis. No differences were found in memory and satisfaction regarding the ICU admission. The experience with the music intervention in the intervention group was scored as “very good” by 12.0%, “good” by 52.0%, and “neutral” by 36.0%. 80.6% of the patients in the intervention group would listen to music during a next hospital admission. Sixty-eight percent of the patients in the control group would listen to music during a next hospital admission. The choice in music varied greatly among patients, but most commonly included pop, Dutch, and classical music.

## Discussion

In this multicenter clinical trial, a music intervention did not decrease anxiety levels in adult ICU patients. Opioid requirement was lower and sleep quality was worse on the first day of the music intervention, but these findings require further research. There were no effects on other (secondary) outcomes, notably no effects on medication use aimed at anxiety reduction (benzodiazepines) or associated outcomes, such as delirium.

The effect of music in the ICU has been a topic of interest in the past decades [16, 19]. The largest RCTs performed in this context by Chlan et al. [20] showed that patient-directed music among ICU patients receiving ventilatory support reduced anxiety. There are several important differences between the study of Chlan et al. and our study. First, the music intervention in Chlan’s study was applied when feeling anxious in contrast to our study that provided the intervention during pre-specified moments. Further, the intervention in our study was aimed to test an immediate result of music on anxiety during 3 days, whereas in Chlan’s study the duration of the intervention was up to 30 days. Chlan et al. did not describe the timing and frequency of anxiety assessment in their study and tested it only once daily. Delirious patients were not excluded from our study when they seemed able to communicate at randomization. These factors might have hampered anxiety assessments in our study. The median daily duration of music intervention in our group was higher, 35.0 (20.8–65.8) vs. 12.0 (0.0–796.0), although the mean durations were longer in Chlan’s trial. Finally, in Chlan’s study a third of the patients dropped out for primary outcome analysis due to less than two VAS assessments. In our study, there was a lower dropout rate of 7% (4/54) in the intervention group and 17% (9/53) in the control group (mostly due to ICU discharge before the intervention). Recent reviews by Bradt et al. [16] and Umbrello et al. [19] concluded that positive effects of music on anxiety could be present in, respectively, mechanically ventilated and ICU patients. However, the RCTs included in these reviews are of low quality. Bradt et al. could perform a meta-analysis for anxiety (VAS and STAI). Pooled analysis (288 patients) resulted in a significant 1.11 lower score in the music group. Quality of the evidence was graded as low and the clinical relevance of 1.11 mean difference is questionable.

In our study, anxiety scores were low, ranging median from 1.5 to 3.0 on the VAS-A difference (compared with a corresponding VAS-A of five in Chlan’s trial). The reason for this is unclear since for example sedation levels were not provided in Chlan’s trial. However, in our trial non-ventilated patients could be included who may experience less anxiety. Patients in our study were included after a median of approximately 9.5 days after ICU admission and 16.5 days after hospital admission, which may have caused habituation to the ICU/hospital environment, and thus levels of anxiety may have dropped at the moment they were included. In the study by Chlan et al. this period was shorter, respectively, 6 (0–40) and 7 (0–33) days for the music and control group. Nevertheless, we hypothesized during the design-phase of the trail that music intervention has a direct effect rather than cumulative or long-term effect, therefore we assessed anxiety directly after applying music for only a limited number of days. Furthermore, because of our hypothesis, our protocol stated that it did not matter what period during the admission the patient would receive the music intervention for it to reduce anxiety. However, given that this was different in our study as compared the study of Chlan et al., where the intervention was applied for a longer period of time, this short application period might in hindsight have been one of the contributing factors to the lack of effect on anxiety in our trial.

We found differences between the groups in sleep quality on the first study day. Also, in a recent meta-analysis by our research group, positive effect of music on sleep quality in the ICU population was found [38]. Furthermore, on the first study day a lower amount of patients required opioids in the music group. This finding seems in line with a meta-analysis in the surgical population [39]. Further research is warranted regarding these outcomes [2, 7]. Importantly, no patient had bad experience with the intervention, which supports the feasibility of the intervention.

### Strengths and limitations

This is the second largest randomized controlled trial studying the effect of music on anxiety in the ICU population following a conventional trial design. However, several limitations should be discussed. This was an un-blinded trial and music intervention did not result in a significant effect possibly due to low baseline anxiety scores, mean initiation after 1 week of ICU admission, and application of only up to 3 days. We chose to not include a control group with headphones without music since the Chlan trial found no difference in effect on anxiety between the headphone only and headphone with patient-directed music groups. In addition, people who listen to music on a daily basis may be more willing in participating in music trials, and this a priori preference could not be easily captured, while it could have influenced the effect of the intervention on anxiety. Also, we hypothesized that the effect of music intervention would be immediate, but given our results, in contrast to the Chlan trial, it cannot be excluded that a music intervention of longer duration might have been more effective due to the repeated exposure over a longer period. Further, the response rates of the anxiety questionnaires, which is dependent on patients’ cognitive ability to score their own anxiety and sleep quality, was challenging since patients admitted to the ICU are often sedated hampering their cognition. Besides, they may experience delirium, and are critically ill which impedes compliance with questionnaires aimed at subjective experiences. Sleep needs further investigation, since sleep was not assessed using a validated tool. The self-made patient experience questionnaire should be mentioned as a limitation, since it is not validated. As mentioned before, the anxiety levels in our study were low. Thus, further decreases of anxiety levels, even when significant, may not be clinically relevant when anxiety seems to be already low. Lastly, the trial started in the middle of the COVID-19 pandemic. This challenged study logistics and might have impeded the quality of the anxiety assessments since adhering to the protocol for this study by nurses was sometimes felt as laborious given the high workload. Furthermore, the time until randomization after ICU admission was significantly delayed in COVID patients (27 vs. 6 days, *p* = 0.0004), which affected the mean delay of music application in the entire study population.

### Clinical implications and future perspectives

This study shows that the previously reported benefit of music intervention on anxiety may not be reproducible and likely depends on setting, exact application method of music intervention and other factors, such as workload of nurses involved in anxiety assessments and the application of the music intervention. Further, the intervention might have a different effect in delirious patients. Clearly, subjective outcome assessments have limitations in the ICU population, since the medical condition and sedation may alter the patient’s responses. We found possible adverse influence of music on sleep quality, which might have been related to the standardized application of the intervention at bedtime rather than being patient-directed. Further studies should focus on factors associated with effectiveness of music intervention and this study and a previous trial provide lessons on how to apply music interventions to be effective. Therefore, we propose music to be administered directly after admission to the ICU, more patient-directed (i.e. make sure the patient has more influence on the timing and ‘dose’ of music administration, e.g. depending on actual feelings of anxiety), and we propose a generally longer and more frequent application during the entire ICU admission. Still, since patients in the intervention group had a good experience with the intervention and music has shown other positive effects in the ICU and other populations, [4, 15, 16, 38, 40] without any side effects, it may still be considered as a useful addition to alleviate suffering of patients, especially upon patients’ request.

## Conclusions

In this clinical trial a targeted 3-day music intervention did not decrease anxiety levels of adult ICU patients who are able to communicate, and did not convincingly affect any other predefined outcomes, which might have been related to low baseline anxiety levels and/or methodological issues related to application of the music intervention. Therefore, further research on effects of music intervention in the critically should take into consideration the methods of application (e.g. regarding timing or patient-incentive in start of the intervention), different outcomes and targets, and selection of patients and baseline anxiety levels in the study population.

### Supplementary Information

**Additional file 1.** CONSORT 2010 checklist of information to include when reporting a randomized trial.

**Additional file 2. **Linear regression statistics anxiety time-by-group interaction.

**Additional file 3.** Anxiety time-by-group interaction.

**Additional file 4.** Secondary outcomes.

**Additional file 5.** Medication requirement.

**Additional file 6.** ICU Memory and experience.

**Additional file 7.** Subgroup analysis on mechanical ventilation.

## Data Availability

The datasets used and/or analysed during the current study are available from the corresponding author on reasonable request.

## Abbreviations

ICU: Intensive care unit
PADIS: Clinical Practice Guidelines for the Prevention and Management of Pain, Agitation/Sedation, Delirium, Immobility, and Sleep Disruption in Adult Patients in the ICU
CONSORT: Consolidated Standards of Reporting Trials
RASS: Richmond agitation-sedation scale
VAS-A: Visual analogue scale for anxiety
STAI-6: State-Trait Anxiety Inventory
CPOT: Critical care pain observation tool
NRS: Numeric Rating Scale
ICDSC: Intensive Care Delirium Screening Checklist
LOS: Length of stay
HR: Heart rate
MAP: Mean arterial pressure
ICUMT: Intensive care unit memory tool
SD: Standard deviation
IQR: Interquartile range
ITT: Intention-to-treat
APACHE: Acute Physiology and Chronic Health Evaluation
