# Supplementary material for: Effect of a music intervention on anxiety in adult critically ill patients: a multicenter randomized clinical trial
Source: J Intensive Care. 2023 Aug 17;11:36. doi: 10.1186/s40560-023-00684-1 (PMC10433648; doi:10.1186/s40560-023-00684-1)
Supplement: Supplementary file 2 — Additional file 2. Linear regression statistics anxiety time-by-group interaction. [file 40560_2023_684_MOESM2_ESM.docx]

**Supplementary file 2 Linear regression statistics anxiety time-by-group interaction**

| Parameter | Estimate | Std. Error | *df* | *t* | Significance | 95% confidence interval | |
| --- | --- | --- | --- | --- | --- | --- | --- |
|  |  |  |  |  |  | **Lower limit** | **Upper limit** |
| Intercept | 3.31 | 2.29 | 85.83 | 1.44 | 0.15 | -1.07 | 7.71 |
| Group | 2.47 | 2.86 | 87.85 | 0.86 | 0.39 | -3.01 | 7.93 |
| Study day | -0.16 | 0.20 | 160.88 | -0.77 | 0.44 | -0.56 | 0.24 |
| Age | -0.01 | 0.03 | 79.87 | -0.21 | 0.84 | -0.07 | 0.06 |
| Sex | 0.43 | 0.66 | 83.32 | 0.66 | 0.51 | -0.82 | 0.06 |
