# Supplementary figures and images for: Effect of a music intervention on anxiety in adult critically ill patients: a multicenter randomized clinical trial
Source: J Intensive Care. 2023 Aug 17;11:36. doi: 10.1186/s40560-023-00684-1 (PMC10433648; doi:10.1186/s40560-023-00684-1)

**Supplementary file 3 Anxiety time-by-group interaction**


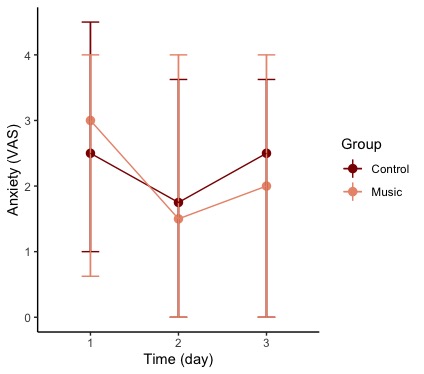

Supplement: Supplementary file 3 — Additional file 3. Anxiety time-by-group interaction. [file 40560_2023_684_MOESM3_ESM.docx]
