# Supplementary material for: Effect of a music intervention on anxiety in adult critically ill patients: a multicenter randomized clinical trial
Source: J Intensive Care. 2023 Aug 17;11:36. doi: 10.1186/s40560-023-00684-1 (PMC10433648; doi:10.1186/s40560-023-00684-1)
Supplement: Supplementary file 4 — Additional file 4. Secondary outcomes. [file 40560_2023_684_MOESM4_ESM.docx]

**Supplementary file 4 Secondary outcomes**

| Outcome | N | Overall  Median/mean (SD/IQR) | N | Control  Median/mean (SD/IQR) | N | Intervention  Median/mean (SD/IQR) | P value |
| --- | --- | --- | --- | --- | --- | --- | --- |
| STAI-6 day 1 | 89 | 44.4 (10.6) | 43 | 44.8 (10.6) | 46 | 44.0 (10.7) | 0.71 |
| STAI-6 day 2 | 83 | 42.5 (11.5) | 39 | 43.1 (11.7) | 44 | 42.0 (11.5) | 0.67 |
| STAI-6 day 3 | 71 | 42.3 (11.3) | 35 | 42.8 (10.5) | 36 | 41.8 (12.2) | 0.73 |
| Sleep day 1 | 82 | 5.0 (3.0-6.0) | 38 | 5.0 (4.0-6.0) | 44 | 4.5 (3.0-5.0) | 0.03 |
| Sleep day 2 | 74 | 4.0 (3.0-5.9) | 34 | 5.0 (3.5-6.0) | 40 | 4.0 (3.0-5.0) | 0.054 |
| Sleep day 3 | 51 | 5.0 (3.3-6.0) | 26 | 5.8 (4.1-6.0) | 25 | 5.0 (3.0-6.0) | 0.13 |
| Pain day 1 | 80 | 0.0 (0.0-0.7) | 35 | 0.0 (0.0-0.7) | 45 | 0.0 (0.0-1.0) | 0.50 |
| Pain day 2 | 77 | 0.0 (0.0-1.0) | 37 | 0.0 (0.0-1.0) | 40 | 0.0 (0.0-1.0) | 0.68 |
| Pain day 3 | 75 | 0.0 (0.0-1.0) | 35 | 0.0 (0.0-0.7) | 40 | 0.0 (0.0-1.6) | 0.21 |
| Pain day 4 | 74 | 0.0 (0.0-1.0) | 34 | 0.0 (0.0-1.0) | 40 | 0.0 (0.0-1.3) | 0.71 |
| Pain day 5 | 66 | 0.2 (0.0-1.0) | 30 | 0.0 (0.0-1.0) | 36 | 0.3 (0.0-1.0) | 0.28 |
| Pain day 6 | 64 | 0.0 (0.0-1.0) | 30 | 0.0 (0.0-1.0) | 34 | 0.0 (0.0-1.0) | 0.57 |
| Pain day 7 | 59 | 0.0 (0.0-0.7) | 27 | 0.0 (0.0-0.5) | 32 | 0.0 (0.0-1.0) | 0.59 |
| ICDSC day 1 | 90 | 1.2 (0.1-2.7) | 42 | 1.2 (0.4-2.5) | 48 | 1.2 (0.0-2.8) | 0.94 |
| Delirium day 1, % | 15 | 16.0 | 7 | 15.9 | 8 | 16.0 | 0.64 |
| ICDSC day 2 | 88 | 1.5 (0.5-2.7) | 41 | 1.5 (0.7-2.7) | 47 | 1.5 (0.5-2.5) | 1.00 |
| Delirium day 2, % | 23 | 24.5 | 11 | 25.0 | 12 | 24.0 | 0.55 |
| ICDSC day 3 | 82 | 1.4 (0.0-2.7) | 37 | 1.3 (0.3-3.0) | 45 | 1.5 (0.0-2.0) | 0.63 |
| Delirium day 3, % | 19 | 20.2 | 9 | 20.5 | 10 | 20.0 | 0.31 |
| ICDSC day 4 | 65 | 1.0 (0.3-2.3) | 31 | 1.0 (0.5-2.7) | 34 | 1.0 (0.0-2.0) | 0.67 |
| Delirium day 4, % | 21 | 22.3 | 10 | 22.7 | 11 | 22.0 | 0.98 |
| ICDSC day 5 | 59 | 1.0 (0.3-2.0) | 29 | 1.0 (0.3-2.7) | 30 | 1.0 (0.1-2.0) | 0.35 |
| Delirium day 5, % | 21 | 22.3 | 12 | 27.3 | 9 | 18.0 | 0.56 |
| ICDSC day 6 | 55 | 1.0 (0.0-2.5) | 29 | 1.0 (0.0-2.5) | 26 | 1.0 (0.4-2.3) | 0.51 |
| Delirium day 6, % | 22 | 23.4 | 10 | 22.7 | 12 | 24.0 | 0.97 |
| ICDSC day 7 | 47 | 1.0 (0.0-2.0) | 21 | 1.0 (0.0-2.0) | 26 | 1.0 (0.1-2.0) | 0.69 |
| Delirium day 7, % | 20 | 21.3 | 10 | 22.7 | 10 | 20.0 | 0.91 |
| HR at baseline* | 94 | 89.9 (17.4) | 44 | 91.9 (16.2) | 50 | 88.0 (19.2) | 0.29 |
| HR day 1 | 94 | 92.7 (12.0) | 44 | 92.4 (11.2) | 50 | 92.9 (12.8) | 0.86 |
| HR day 2 | 91 | 96.0 (90.5-100.0) | 42 | 96.3 (88.5-103.4) | 49 | 96.0 (91.0-99.0) | 0.64 |
| HR day 3 | 85 | 95.8 (14.0) | 39 | 97.3 (14.6) | 46 | 94.5 (13.5) | 0.42 |
| HR day 4 | 86 | 92.9 (16.0) | 41 | 97.0 (18.8) | 45 | 89.2 (12.1) | 0.03 |
| HR day 5 | 81 | 91.6 (16.1) | 39 | 92.9 (17.2) | 42 | 90.3 (15.1) | 0.47 |
| HR day 6 | 79 | 91.0 (14.1) | 38 | 93.1 (14.2) | 41 | 88.9 (13.8) | 0.19 |
| HR day 7 | 75 | 89.9 (15.7) | 34 | 93.0 (17.5) | 41 | 87.4 (13.7) | 0.13 |
| MAP at baseline^*^ | 93 | 85.4 (12.8) | 44 | 85.3 (12.8) | 49 | 85.6 (13.1) | 0.91 |
| MAP day 1 | 94 | 85.5 (12.9) | 44 | 87.5 (12.2) | 50 | 83.7 (13.3) | 0.16 |
| MAP day 2 | 91 | 89.0 (78.3-97.3) | 42 | 88.5 (76.1-100.3) | 49 | 91.0 (80.0-97.0) | 0.83 |
| MAP day 3 | 84 | 88.4 (13.6) | 39 | 90.0 (14.4) | 45 | 86.9 (12.8) | 0.31 |
| MAP day 4 | 86 | 87.3 (11.5) | 41 | 87.0 (11.2) | 45 | 87.5 (12.0) | 0.86 |
| MAP day 5 | 81 | 84.9 (11.3) | 39 | 85.1 (12.5) | 42 | 84.7 (10.1) | 0.88 |
| MAP day 6 | 79 | 84.5 (74.8-90.3) | 38 | 87.3 (78.3-92.8) | 41 | 82.5 (74.5-89.0) | 0.19 |
| MAP day 7 | 75 | 85.9 (12.4) | 34 | 86.2 (13.3) | 41 | 85.6 (11.8) | 0.84 |
| RASS day 1 | 88 | 0 (-1 - 0) | 42 | 0 (0-0) | 46 | 0 (-1 - 0) | 0.13 |
| RASS day 2 | 85 | 0 (0-0) | 39 | 0 (0-0) | 46 | 0 (0-0) | 0.83 |
| RASS day 3 | 79 | 0 (0-0) | 37 | 0 (-1 - 0) | 42 | 0 (0-0) | 0.56 |
| RASS day 4 | 61 | 0 (0-0) | 29 | 0 (0-0) | 32 | 0 (-1 - 0) | 0.13 |
| RASS day 5 | 55 | 0 (-1 - 0) | 29 | 0 (-1 - 0) | 26 | 0 (0-0) | 0.52 |
| RASS day 6 | 45 | 0 (0-0) | 26 | 0 (0-0) | 19 | 0 (0-0) | 0.56 |
| RASS day 7 | 40 | 0 (0-0) | 23 | 0 (0-0) | 17 | 0 (0-0) | 0.64 |
| Hospital LOS after inclusion, days | 89 | 21.0 (14.0-32.0) | 40 | 21.0 (16.8-40.3) | 49 | 21.0 (12.0-30.0) | 0.14 |
| ICU LOS after inclusion, days | 92 | 9.5 (4.0-17.0) | 42 | 10.0 (6.0-17.8) | 50 | 8.0 (4.0-12.0) | 0.21 |
| MV duration total, hours | 94 | 198.9 (11.2-685.7) | 44 | 232.2 (29.4-757.7) | 50 | 178.4 (0.0-452.9) | 0.26 |
| Mortality within 30 days after inclusion, N, % | 17 | 18.1 | 4 | 9.1 | 13 | 26.0 | 0.06 |
| Complications, N, % | 19 | 20.2 | 6 | 13.6 | 13 | 26.0 | 0.22 |
| - Self-extubation, n, % | 5 | 23.8 | 2 | 40.0 | 3 | 60.0 | 0.78 |
| - Removal line/tube, n, % | 11 | 52.4 | 3 | 27.3 | 8 | 72.7 |  |
| - Other^a^, n, % | 5 | 23.8 | 1 | 20.0 | 4 | 80.0 |  |
| N; number of patients, n; number of events, SD; standard deviation, IQR; interquartile range, ICDSC; intensive care delirium screening checklist, HR; heart rate, MAP; mean arterial pressure, RASS; Richmond agitation-sedation scale, LOS; length of stay; ICU; intensive care unit, MV; mechanical ventilation  ^*^Baseline is defined as day 0, the day before the intervention started.  ^a^Other; hallucinations (4 patients), panic (1 patient).  Based on the Shapiro-Wilk test of normality means (SD) or medians (IQR) were reported. | | | | | | | |
