# Supplementary material for: Effect of a music intervention on anxiety in adult critically ill patients: a multicenter randomized clinical trial
Source: J Intensive Care. 2023 Aug 17;11:36. doi: 10.1186/s40560-023-00684-1 (PMC10433648; doi:10.1186/s40560-023-00684-1)
Supplement: Supplementary file 5 — Additional file 5. Medication requirement. [file 40560_2023_684_MOESM5_ESM.docx]

**Supplementary file 5 Medication requirement**

|  | Overall | | Control | | | Intervention | | |  | |
| --- | --- | --- | --- | --- | --- | --- | --- | --- | --- | --- |
| Drug, N/mean, %/SD | N | % | | N | % | | N | % | | P^a^ |
| Continuous sedatives at baseline^b^ | 20 | 21.3 | | 10 | 22.7 | | 10 | 20.0 | | 0.75 |
| Continuous Sedatives day 1 | 20 | 21.3 | | 11 | 25.0 | | 9 | 18.0 | | 0.57 |
| Continuous Sedatives day 2 | 12 | 12.8 | | 7 | 15.9 | | 5 | 10.0 | | 0.58 |
| Continuous Sedatives day 3 | 11 | 11.7 | | 6 | 13.6 | | 5 | 10.0 | | 0.82 |
| Continuous Sedatives day 4 | 10 | 10.6 | | 4 | 9.1 | | 6 | 12.0 | | 0.90 |
| Continuous Sedatives day 5 | 11 | 11.7 | | 6 | 13.6 | | 5 | 10.0 | | 0.82 |
| Continuous Sedatives day 6 | 10 | 10.6 | | 5 | 11.4 | | 5 | 10.0 | | 1.00 |
| Continuous Sedatives day 7 | 10 | 10.6 | | 3 | 6.8 | | 7 | 14.0 | | 0.43 |
| Propofol at baseline | 257.6 (981.6) | | | 289.4 (1174.0) | | | 229.7 (785.5) | | | 0.95 |
| Propofol day 1 | 160.6 (663.8) | | | 189.4 (640.3) | | | 135.3 (689.4) | | | 0.81 |
| Propofol day 2 | 113.9 (666.1) | | | 89.7 (595.2) | | | 135.3 (728.1) | | | 0.65 |
| Propofol day 3 | 76.4 (498.7) | | | 57.3 (270.9) | | | 93.2 (638.0) | | | 0.91 |
| Propofol day 4 | 93.0 (638.0) | | | 0.0 (0.0) | | | 174.8 (870.6) | | | 0.10 |
| Propofol day 5 | 115.9 (658.4) | | | 50.6 (233.2) | | | 173.3 (876.2) | | | 0.59 |
| Propofol day 6 | 185.8 (919.4) | | | 95.1 (593.5) | | | 265.6 (1132.0) | | | 0.75 |
| Propofol day 7 | 178.7 (785.1) | | | 71.6 (474.9) | | | 272.9 (976.0) | | | 0.13 |
| Clonidine at baseline | 0.10 (0.33) | | | 0.09 (0.31) | | | 0.11 (0.35) | | | 0.88 |
| Clonidine day 1 | 0.09 (0.30) | | | 0.08 (0.26) | | | 0.10 (0.33) | | | 0.90 |
| Clonidine day 2 | 0.08 (0.28) | | | 0.07 (0.25) | | | 0.09 (0.30) | | | 0.91 |
| Clonidine day 3 | 0.06 (0.22) | | | 0.06 (0.24) | | | 0.06 (0.19) | | | 0.86 |
| Clonidine day 4 | 0.05 (0.20) | | | 0.07 (0.25) | | | 0.03 (0.14) | | | 0.53 |
| Clonidine day 5 | 0.04 (0.18) | | | 0.06 (0.23) | | | 0.02 (0.12) | | | 0.52 |
| Clonidine day 6 | 0.03 (0.14) | | | 0.04 (0.18) | | | 0.01 (0.09) | | | 0.26 |
| Clonidine day 7 | 0.01 (0.07) | | | 0.00 (0.03) | | | 0.02 (0.09) | | | 0.64 |
| Midazolam at baseline | NA | | | NA | | | NA | | | NA |
| Midazolam day 1 | 0.05 (0.52) | | | 0.11 (0.75) | | | 0.00 (0.00) | | | 0.30 |
| Midazolam day 2 | 0.05 (0.52) | | | 0.11 (0.75) | | | 0.00 (0.00) | | | 0.30 |
| Midazolam day 3 | NA | | | NA | | | NA | | | NA |
| Midazolam day 4 | 0.05 (0.52) | | | 0.00 (0.00) | | | 0.10 (0.71) | | | 0.36 |
| Midazolam day 5 | 0.03 (0.26) | | | 0.00 (0.00) | | | 0.05 (0.35) | | | 0.36 |
| Midazolam day 6 | 1.2 (10.5) | | | 0.00 (0.00) | | | 2.23 (14.36) | | | 0.19 |
| Midazolam day 7 | 0.63 (5.17) | | | 0.00 (0.00) | | | 1.18 (7.07) | | | 0.19 |
| Dexmedetomidine at baseline | 0.13 (1.29) | | | 0.28 (1.88) | | | 0.00 (0.00) | | | 0.30 |
| Dexmedetomidine day 1 | 0.09 (0.91) | | | 0.20 (1.33) | | | 0.00 (0.00) | | | 0.30 |
| Dexmedetomidine day 2 | 0.08 (0.74) | | | 0.16 (1.09) | | | 0.00 (0.00) | | | 0.30 |
| Dexmedetomidine day 3 | 0.12 (0.84) | | | 0.16 (1.09) | | | 0.08 (0.54) | | | 0.93 |
| Dexmedetomidine day 4 | 0.23 (1.69) | | | 0.16 (1.09) | | | 0.30 (2.09) | | | 0.95 |
| Dexmedetomidine day 5 | 0.29 (2.23) | | | 0.16 (1.09) | | | 0.41 (2.89) | | | 0.95 |
| Dexmedetomidine day 6 | 0.35 (2.72) | | | 0.16 (1.09) | | | 0.51 (3.59) | | | 0.95 |
| Dexmedetomidine day 7 | 0.34 (2.62) | | | 0.16 (1.09) | | | 0.49 (3.46) | | | 0.95 |
| Opioids at baseline | 50 | 53.2 | | 28 | 63.6 | | 22 | 22.0 | | 0.06 |
| Opioids day 1 | 50 | 53.2 | | 29 | 65.9 | | 21 | 42.0 | | 0.02 |
| Opioids day 2 | 42 | 44.6 | | 24 | 54.5 | | 18 | 36.0 | | 0.07 |
| Opioids day 3 | 41 | 43.6 | | 22 | 50.0 | | 19 | 39.0 | | 0.24 |
| Opioids day 4 | 39 | 41.4 | | 21 | 47.7 | | 18 | 36.0 | | 0.25 |
| Opioids day 5 | 34 | 36.2 | | 19 | 43.2 | | 15 | 30.0 | | 0.18 |
| Opioids day 6 | 34 | 36.2 | | 18 | 40.9 | | 16 | 32.0 | | 0.37 |
| Opioids day 7 | 34 | 36.2 | | 17 | 38.6 | | 18 | 36.0 | | 0.79 |
| Fentanyl equivalents^c^ at baseline | 2.2 (4.4) | | | 2.7 (4.7) | | | 1.7 (4.2) | | | 0.04 |
| Fentanyl equivalents day 1 | 2.4 (5.3) | | | 3.1 (5.7) | | | 1.8 (5.0) | | | 0.70* |
| Fentanyl equivalents day 2 | 1.9 (4.9) | | | 2.3 (4.7) | | | 1.5 (5.1) | | | 0.59* |
| Fentanyl equivalents day 3 | 1.5 (4.3) | | | 1.8 (4.4) | | | 1.3 (4.3) | | | 0.42* |
| Fentanyl equivalents day 4 | 1.5 (4.4) | | | 1.8 (4.5) | | | 1.3 (4.4) | | | 0.36* |
| Fentanyl equivalents day 5 | 1.4 (4.1) | | | 1.6 (3.8) | | | 1.3 (4.3) | | | 0.40* |
| Fentanyl equivalents day 6 | 1.2 (3.8) | | | 0.8 (2.3) | | | 1.5 (4.8) | | | 0.73* |
| Fentanyl equivalents day 7 | 1.2 (4.1) | | | 0.7 (2.0) | | | 1.7 (5.3) | | | 0.70* |
| Antipsychotics at baseline | 38 | 40.4 | | 14 | 31.8 | | 24 | 48.0 | | 0.11 |
| Antipsychotics day 1 | 44 | 46.8 | | 18 | 40.9 | | 26 | 52.0 | | 0.28 |
| Antipsychotics day 2 | 43 | 45.7 | | 19 | 43.2 | | 24 | 48.0 | | 0.53 |
| Antipsychotics day 3 | 46 | 48.9 | | 20 | 45.5 | | 26 | 52.0 | | 0.47 |
| Antipsychotics day 4 | 41 | 43.6 | | 18 | 40.9 | | 23 | 46.0 | | 0.80 |
| Antipsychotics day 5 | 42 | 44.6 | | 20 | 45.5 | | 22 | 44.0 | | 0.39 |
| Antipsychotics day 6 | 39 | 41.4 | | 19 | 43.2 | | 20 | 40.0 | | 0.12 |
| Antipsychotics day 7 | 36 | 38.3 | | 16 | 36.4 | | 20 | 40.0 | | 0.11 |
| Haloperidol at baseline | 1.56 (3.37) | | | 1.48 (3.23) | | | 1.63 (3.52) | | | 0.79 |
| Haloperidol day 1 | 1.65 (3.49) | | | 1.82 (3.54) | | | 1.50 (3.47) | | | 0.64 |
| Haloperidol day 2 | 1.91 (3.77) | | | 2.20 (3.96) | | | 1.65 (3.61) | | | 0.39 |
| Haloperidol day 3 | 1.98 (3.98) | | | 1.98 (3.74) | | | 1.99 (4.21) | | | 0.88 |
| Haloperidol day 4 | 1.82 (3.99) | | | 1.41 (3.29) | | | 2.19 (4.51) | | | 0.41 |
| Haloperidol day 5 | 2.01 (4.02) | | | 1.64 (2.97) | | | 2.45 (4.76) | | | 0.71 |
| Haloperidol day 6 | 1.80 (3.80) | | | 1.25 (2.31) | | | 2.28 (4.72) | | | 0.75 |
| Haloperidol day 7 | 1.37 (3.18) | | | 1.06 (2.27) | | | 1.65 (3.80) | | | 0.57 |
| Quetiapine at baseline | 7.45 (15.48) | | | 6.53 (15.13) | | | 8.25 (15.89) | | | 0.53 |
| Quetiapine day 1 | 7.73 (14.32) | | | 7.67 (14.06) | | | 7.78 (14.69) | | | 0.86 |
| Quetiapine day 2 | 8.99 (15.49) | | | 8.97 (16.25) | | | 9.00 (14.85) | | | 0.76 |
| Quetiapine day 3 | 11.04 (19.04) | | | 11.65 (20.95) | | | 10.50 (17.38) | | | 0.90 |
| Quetiapine day 4 | 9.57 (20.61) | | | 13.07 (26.41) | | | 6.50 (13.18) | | | 0.27 |
| Quetiapine day 5 | 9.04 (17.72) | | | 10.80 (20.28) | | | 7.50 (15.15) | | | 0.47 |
| Quetiapine day 6 | 8.78 (17.85) | | | 10.80 (20.64) | | | 7.00 (14.97) | | | 0.46 |
| Quetiapine day 7 | 6.78 (14.85) | | | 7.95 (16.64) | | | 5.75 (13.17) | | | 0.78 |
| Olanzapine at baseline | 0.12 (1.03) | | | 0.00 (0.00) | | | 0.20 (1.41) | | | 0.36 |
| Olanzapine day 1 | 0.13 (0.77) | | | 0.00 (0.00) | | | 0.25 (1.04) | | | 0.10 |
| Olanzapine day 2 | 0.05 (0.36) | | | 0.00 (0.00) | | | 0.1 (0.49) | | | 0.19 |
| Olanzapine day 3 | 0.03 (0.26) | | | 0.00 (0.00) | | | 0.06 (0.36) | | | 0.19 |
| Olanzapine day 4 | 0.08 (0.57) | | | 0.00 (0.00) | | | 0.15 (0.78) | | | 0.19 |
| Olanzapine day 5 | 0.08 (0.57) | | | 0.00 (0.00) | | | 0.15 (0.78) | | | 0.19 |
| Olanzapine day 6 | 0.11 (0.63) | | | 0.06 (0.38) | | | 0.15 (0.78) | | | 0.64 |
| Olanzapine day 7 | 0.11 (0.63) | | | 0.06 (0.38) | | | 0.15 (0.78) | | | 0.64 |
| Intermittent sedatives at baseline | 30 | 31.9 | | 13 | 29.5 | | 17 | 34.0 | | 0.64 |
| Intermittent sedatives day 1 | 29 | 30.9 | | 12 | 27.3 | | 17 | 34.0 | | 0.48 |
| Intermittent sedatives day 2 | 35 | 37.2 | | 13 | 29.5 | | 22 | 44.0 | | 0.22 |
| Intermittent sedatives day 3 | 35 | 37.2 | | 15 | 34.1 | | 20 | 40.0 | | 0.47 |
| Intermittent sedatives day 4 | 29 | 30.9 | | 14 | 14.9 | | 15 | 30.0 | | 0.81 |
| Intermittent sedatives day 5 | 28 | 29.8 | | 13 | 29.5 | | 15 | 30.0 | | 0.44 |
| Intermittent sedatives day 6 | 28 | 29.8 | | 11 | 25.0 | | 17 | 34.0 | | 0.16 |
| Intermittent sedatives day 7 | 23 | 24.5 | | 10 | 22.7 | | 13 | 26.0 | | 0.11 |
| Lorazepam equivalents at baseline | 0.82 (1.77) | | | 0.82 (1.77) | | | 0.82 (1.78) | | | 0.93 |
| Lorazepam equivalents day 1 | 0.87 (1.73) | | | 0.67 (1.47) | | | 1.03 (1.93) | | | 0.36 |
| Lorazepam equivalents day 2 | 0.95 (1.67) | | | 0.87 (1.77) | | | 1.03 (1.59) | | | 0.27 |
| Lorazepam equivalents day 3 | 1.0 (1.78) | | | 0.94 (1.84) | | | 1.05 (1.75) | | | 0.48 |
| Lorazepam equivalents day 4 | 0.81 (1.59) | | | 0.88 (1.84) | | | 0.75 (1.36) | | | 0.98 |
| Lorazepam equivalents day 5 | 0.87 (1.84) | | | 0.89 (1.93) | | | 0.85 (1.78) | | | 0.98 |
| Lorazepam equivalents day 6 | 0.81 (1.61) | | | 0.75 (1.77) | | | 0.86 (1.48) | | | 0.38 |
| Lorazepam equivalents day 7 | 0.60 (1.29) | | | 0.55 (1.27) | | | 0.65 (1.32) | | | 0.68 |
| For sedatives, opioids, antipsychotics, and benzodiazepines the number of patients that received these are reported, then these were specified by analyzing the dosages.  N; number of patients, SD; standard deviation, NA; not applicable.  ^*^Corrected for baseline difference in fentanyl equivalents.  ^a^Means and SD’s are reported but tested with Wilcoxon rank sum test.  ^b^Baseline is defined as day 0, the day before the intervention started.  ^c^Calculations of equivalents are described in the manuscript under statistical analysis.  Based on the Shapiro-Wilk test of normality means (SD) or medians (IQR) were reported. | | | | | | | | | | |
