# Supplementary material for: Effect of a music intervention on anxiety in adult critically ill patients: a multicenter randomized clinical trial
Source: J Intensive Care. 2023 Aug 17;11:36. doi: 10.1186/s40560-023-00684-1 (PMC10433648; doi:10.1186/s40560-023-00684-1)
Supplement: Supplementary file 6 — Additional file 6. ICU Memory and experience. [file 40560_2023_684_MOESM6_ESM.docx]

**Supplementary file 6 ICU Memory and experience**

|  | N | Overall | N | Control | N | Intervention | P |
| --- | --- | --- | --- | --- | --- | --- | --- |
| Memory | | | | | | | |
| Hospital admission | 38  9  17 | Clear  Vague  No memory at all | 20  3  9 | Clear  Vague  No memory at all | 18  6  8 | Clear  Vague  No memory at all | 0.56 |
| Hospital before ICU admission | 18  24  17  5 | Everything  Partly  Nothing  NA | 10  13  7  2 | Everything  Partly  Nothing  NA | 8  11  10  3 | Everything  Partly  Nothing  NA | 0.77 |
| ICU stay | 47  17 | Yes  No | 22  10 | Yes  No | 25  7 | Yes  No | 0.57 |
| Remembered items, n | 68 | 7.0 (4.0-12.0) | 32 | 7.0 (3.0-11.5 | 32 | 9.0 (6.8-13.0) | 0.11 |
| Transfer from ICU to nursing department | 46  10  5  2  1 | Clear  Vague  None  NA  missing | 21  7  1  2  1 | Clear  Vague  None  NA  missing | 25  3  4 | Clear  Vague  None | 0.20 |
| Forced memories^a^ | 20  44 | Yes  No | 8  24 | Yes  No | 12  20 | Yes  No | 0.42 |
| Admission discussed with people, n | 68 | 2.0 (1.0-3.3) | 32 | 2.0 (1.0-3.0) | 32 | 2.0 (1.0-4.0) | 0.89 |
| Experience | | | | | | | |
| ICU satisfaction score, median (IQR) | 68 | 8.0 (7.0-9.25) | 32 | 8.0 (4.0-9.0) | 32 | 8.0 (7.0-10.0 | 0.14 |
| ICU satisfaction, Likert-scale | 19  32  4  2  0 | Very good  Good  Neutral (did not matter)  Bad  Very bad | 7  18  2  2  0 | Very good  Good  Neutral (did not matter)  Bad  Very bad | 12  14  2  0  0 | Very good  Good  Neutral (did not matter)  Bad  Very bad | 0.28 |
| Would listen to music next admission | 31 | NA | 21  9  1 | Yes  No  Does not know | NA | NA | NA |
| Experience music intervention | 25 | NA | NA | NA | 3  13  9 | Very good  Good  Neutral | NA |
| Listen to music again | 31 | NA | NA | NA | 25  6 | Yes  No | NA  NA |
| Type of music^b^ | 31 | NA | NA | NA | 9  7  5  27 | Pop  Dutch  Classic  Other | NA |
| N; number of patients, ICU; intensive care unit, IQR; interquartile range, NA; not applicable, n = number of items/people  ^a^Forced memories are defined as memories which the patients did not want to recall, but was not able to.  ^b^Type of music “Other”: soul, jazz, Arabic, religious, etc. | | | | | | | |
