# Supplementary material for: Effect of a music intervention on anxiety in adult critically ill patients: a multicenter randomized clinical trial
Source: J Intensive Care. 2023 Aug 17;11:36. doi: 10.1186/s40560-023-00684-1 (PMC10433648; doi:10.1186/s40560-023-00684-1)
Supplement: Supplementary file 7 — Additional file 7. Subgroup analysis on mechanical ventilation. [file 40560_2023_684_MOESM7_ESM.docx]

**Supplementary file 7 Subgroup analysis on mechanical ventilation**

| Mechanical ventilation group | | | | | | |
| --- | --- | --- | --- | --- | --- | --- |
| Day | 1 | | 2 | | 3 | |
|  | Control | Music | Control | Music | Control | Music |
| N | 34 | 36 | 31 | 31 | 30 | 30 |
| Median | 2.5 | 3.25 | 2.5 | 1.0 | 2.5 | 2.0 |
| IQR | 1-4.5 | 1-4.63 | 1.23-4.25 | 0-4.5 | 0-3.88 | 0.13-4.63 |
| P | 0.70 | | 0.37 | | 0.92 | |
| No mechanical ventilation group | | | | | | |
| N | 10 | 14 | 9 | 14 | 6 | 9 |
| Median | 2.75 | 3.0 | 0 | 2 | 1.75 | 1.5 |
| IQR | 0.25-4.63 | 0.5-3.0 | 0-2 | 1-3.75 | 0.38-2.38 | 0-4 |
| P | 0.86 | | 0.08 | | 1.00 | |
| N= number of patients; IQR = interquartile range  The Wilcoxon Rank-Sum test was used to test significancy. | | | | | | |
